# Automated detection of fatal cerebral haemorrhage in postmortem CT data

**DOI:** 10.1007/s00414-024-03183-6

**Published:** 2024-02-08

**Authors:** Andrea Zirn, Eva Scheurer, Claudia Lenz

**Affiliations:** 1 Institute of Forensic Medicine, Department of Biomedical Engineering, University of Basel, Pestalozzistrasse 22, 4056 Basel, Switzerland; 2 Institute of Forensic Medicine, Health Department Basel-Stadt, Basel, Switzerland

**Keywords:** Computed tomography, PMCT, Artificial intelligence, Postmortem, Cause of death, Cerebral haemorrhage

## Abstract

During the last years, the detection of different causes of death based on postmortem imaging findings became more and more relevant. Especially postmortem computed tomography (PMCT) as a non-invasive, relatively cheap, and fast technique is progressively used as an important imaging tool for supporting autopsies. Additionally, previous works showed that deep learning applications yielded robust results for in vivo medical imaging interpretation. In this work, we propose a pipeline to identify fatal cerebral haemorrhage on three-dimensional PMCT data. We retrospectively selected 81 PMCT cases from the database of our institute, whereby 36 cases suffered from a fatal cerebral haemorrhage as confirmed by autopsy. The remaining 45 cases were considered as neurologically healthy. Based on these datasets, six machine learning classifiers (*k*-nearest neighbour, Gaussian naive Bayes, logistic regression, decision tree, linear discriminant analysis, and support vector machine) were executed and two deep learning models, namely a convolutional neural network (CNN) and a densely connected convolutional network (DenseNet), were trained. For all algorithms, 80% of the data was randomly selected for training and 20% for validation purposes and a five-fold cross-validation was executed. The best-performing classification algorithm for fatal cerebral haemorrhage was the artificial neural network CNN, which resulted in an accuracy of 0.94 for all folds. In the future, artificial neural network algorithms may be applied by forensic pathologists as a helpful computer-assisted diagnostics tool supporting PMCT-based evaluation of cause of death.

## Introduction

Over the last decades, postmortem imaging has shown remarkable growth. Especially postmortem computed tomography (PMCT) is nowadays a well-established non-invasive imaging method, which is routinely applied in many forensic institutes [1]. The main purposes of PMCT are to provide evidence, to support diagnosis, to help interpretation, to guide the autopsy, and in some rare cases to replace autopsy [2]. However, the possibilities regarding the detection, interpretation, and visualization of relevant forensic findings are still under evaluation [3]. In comparison to the application of PMCT and autopsy together, performing only an autopsy shows limitations, as it is subject- and observer-dependent [3], and shows impeded detection of intraventricular haemorrhages [1].

In the existing publications, PMCT pathologies were mainly either detected by trained radiologists or based on the Hounsfield unit (HU) profile of the CT images [1]. Tappero et al. [4] successfully demonstrated that extraaxial and intracranial haemorrhages can be identified and located in PMCT, even in decomposed cases. The subsequent examination by autopsy was able to confirm these findings, but had difficulties to locate the intracranial bleedings due to the liquid state of the brain [4]. In all cases, the haemorrhages were interpreted as potentially fatal due to the extent and location. Moreover, the study of Añon et al. [5] achieved an accuracy of 89% for the detection of haemorrhages in traumatic brain injury. The group of Graziani et al. [6] could confirm these findings and also found a high level of agreement between autopsy and PMCT findings for skull fractures, brain oedema, bullet trajectories, and the presence of gas in tissues or cavities. Additionally, Femia et al. [7] demonstrated that there was a 100% overlap of findings at autopsy versus PMCT for haemopericardium, intracranial subarachnoid haemorrhage, and head trauma with fractures or haematomas. In an extensive study from Kasahara et al. [8], 339 cases with PMCT data, in which the cause of death (COD) was detected by autopsy, were reviewed. Each PMCT dataset was categorized into one of these three categories: diagnosable, suggestive (COD was suggested by PMCT findings), and non-diagnosable. Only 25 cases were categorized into diagnosable and 183 cases into suggestive. Cases that fell into the category diagnosable had findings of trauma, head injury, and thoracic injury, especially intracranial haematoma, endogenous cerebral haemorrhage, and traumatic cardiac rupture. These results demonstrated that even though PMCT can detect signs of certain injuries or causes of death, those findings are mostly not sufficient to determine the COD in most cases without any further autoptic investigations.

Several researchers have developed machine learning (ML) algorithms to automate identification of pathologies, but so far mostly using in vivo CT imaging [9–11]. Chilamkurthy et al. [10] developed and validated a set of deep learning algorithms for automated detection of intracranial, intraparenchymal, intraventricular, subdural, extradural and subarachnoid haemorrhages, and calvarial fractures on ante mortem CT images. Their algorithm based on a residual network (ResNet) [12] achieved an area under the curve (AUC) of over 0.9 for detecting all types of haemorrhage. The group of Jnawali [9] also implemented an automated deep learning framework, which learned to detect brain haemorrhage and achieved AUCs of 0.87. Another worth mentioning work from Li et al. [11] described a Slice Dependency Learning Model (SDLM), which used a series of ante mortem brain CT images and slice dependencies between different slices to predict abnormalities.

The goal of this work was to perform binary classifications for automatically detecting fatal cranial haemorrhages on postmortem CT data. Thereby, state-of-the-art machine and deep learning algorithms were evaluated and compared to each other. Additionally, the value of PMCT imaging as a classification tool for cerebral haemorrhages as the COD was evaluated.

## Material and methods

### PMCT data acquisition

All CT datasets included in this study were selected from the database of our institute and were generated during the period of 2011 to 2020. They were acquired using the CT system Siemens SOMATOM Emotion 16 slice scanner with a tube voltage of 130 kV. The protocol for the head had a slice thickness of 1.5 mm and the convolution kernel H10S was applied. The 3D reconstructions were generated from the CT data with a reconstruction matrix of 512 × 512 × 799 pixels with a final spacing of 1 mm on each plane for the head.

### Included PMCT datasets

The COD was defined according to the autopsy findings as described in the reports by the forensic pathologists and served as ground truth for the definition of the COD throughout this work. Thereby, cases with fatal cerebral haemorrhage were labelled as pathological and data with a heart failure as COD were used as neurologically healthy controls. The exclusion criteria for both groups were the age at death, which had to be greater than 17 years, visible alterations due to head trauma, or advanced decomposition. In the given period from 2011 to 2022, 46 cases of fatal cerebral haemorrhage were found in the database. Nine of these cases had to be excluded due to image artefacts and one due to the minimal required age. The cases for the healthy control group were selected to ensure a similar distribution of age, sex, and postmortem interval. Finally, a total of 81 cases, 36 with fatal cerebral haemorrhages and 45 healthy control cases, were included in this study. The final pathological group presented a total number of 15 intracerebral haemorrhages, six epidural haemorrhages, nine subdural haemorrhages, and 11 subarachnoid haemorrhages. Thereby, five subjects showed two types of haemorrhages, whereas 31 subjects showed only one type. In the pathological group, the mean age at death was 64.8 years (± 18.8 years, range from 32 to 101 years) with 15 female and 21 male subjects. The mean postmortem interval for this group was 42 h (± 30.8 h, range from 7 to 144 h). In addition, the neurologically healthy control cases without cerebral haemorrhage consisted of 45 (15 female and 30 male) subjects with a mean age at death of 64.5 years (± 14.4 years, range from 29 to 98 years). For this group, the mean postmortem interval was 35 h (± 27.9 h, range from 4 to 96 h).

### PMCT data processing

The first step of the data processing was the conversion from Digital Imaging and Communications in Medicine (DICOM) to The Neuroimaging Informatics Technology Initiative (NIFTI) image format to prepare the data for segmentation, as the NIFTI format is less complex than DICOM [13]. Furthermore, the data was manually cropped to the size 420 × 420 × 420 pixels (px) to include the region of interest, which only contained the head without the neck area.

**Fig. 1:**
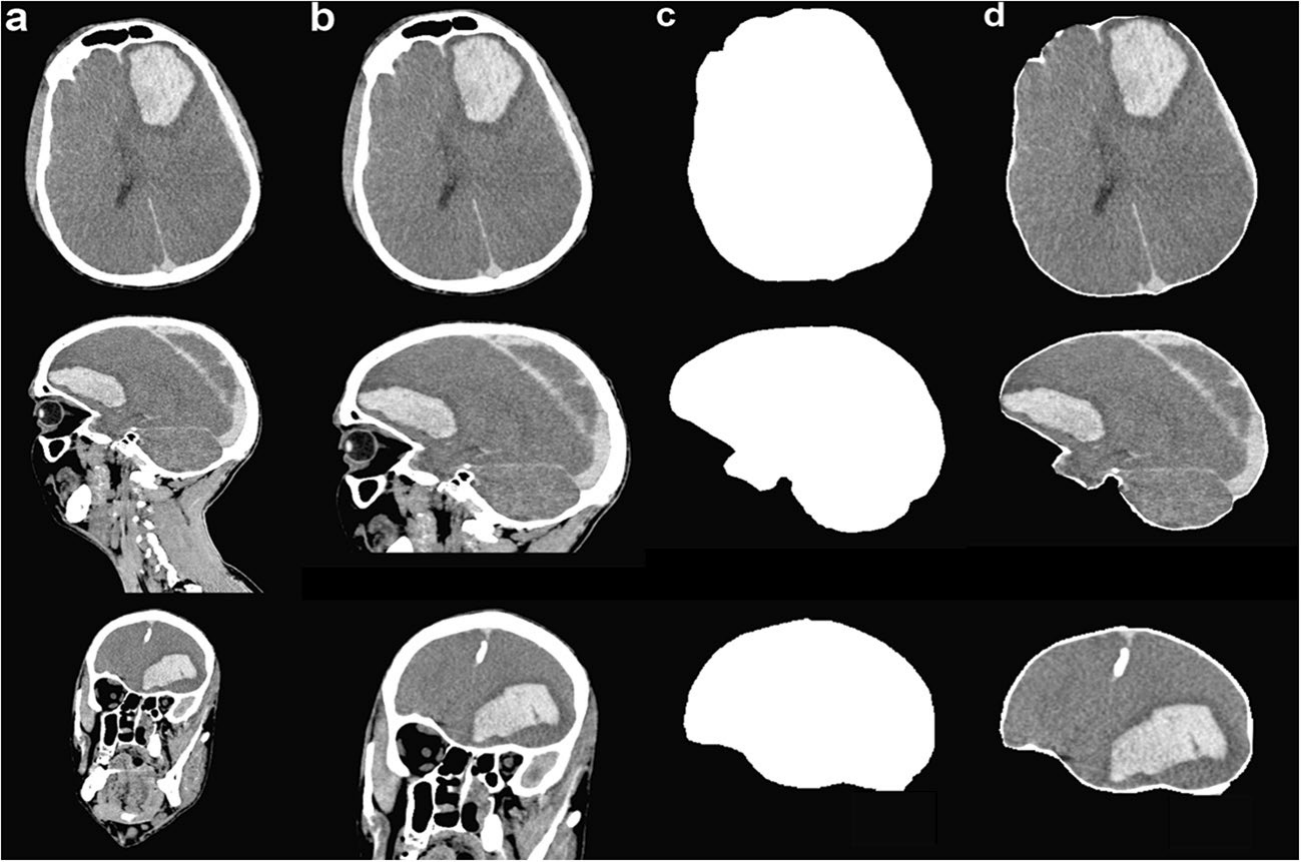
Exemplary visualization of the image segmentation process. Top: axial plane, middle: sagittal plane, bottom: coronal plane. **a** Original CT images (windowed), **b** cropped images with size 420 × 420 × 420 px, **c** mask created by FSL, and **d** segmented brain images

For the cranial segmentation, the fully automated segmentation based on the functional MRI of the brain (FMRIB) software library (FSL) [14] was used. The FSL command-line bash script (FSL-Muschelli) can be downloaded from GitHub [15]. Following the refined process from W. Breakey et al. [16], which showed fast and accurate results using CT images, a threshold was applied to all images using a range of 5 to 100 HU and smoothed using a 3D Gaussian kernel of 1 (*σ* = 1 mm^3^) and a fractional intensity of 0.35. The code performs a cranial segmentation, whereas the output of the pipeline is a binary segmentation mask and a dataset illustrating only the brain inside the skull, as also demonstrated by Bauer et al. [17]. Since the FSL algorithm did perform some image processing (normalization, Gaussian blurring) on the CT images to support the segmentation process, the original values (in HU) were modified. To preserve the original values for our data analysis, every unprocessed CT image was multiplied with the created binary mask image using an in house Python (version 3.10) [18] code, specifically implemented for this study. The final result was a dataset with original HU values only containing values of the brain inside the skull. All datasets were then saved to the NIFTI format using the Python library nibabel (version 3.2.2) [19]. The different steps of the image segmentation process are visualized in Fig. 1.

### PMCT exploratory analysis

The aim of the exploratory data analysis was to perform initial investigations on the data to discover potential patterns or anomalies with the help of graphical representations. For each dataset of both categories, the occurrence of each unique HU value was counted and the average occurrence of these values within the same category (e.g., cerebral haemorrhage) was analysed.

### PMCT data classification

For the data to be used for machine learning and to save computational time, the images were further adjusted to the resolution 128 × 128 × 128 px. Additionally, normalization was performed with a window centre of 40 and window width of 80 [13]. After these steps, all data was manually checked to ensure whole brain coverage. These pre-processing steps aimed to eliminate variations in pixel intensity and size, enabling the machine learning algorithms to operate consistently and effectively across the datasets. For each dataset, a label was applied, which was 1 for pathological cases and 0 for neurologically healthy cases. All the data and labels were then shuffled before splitting them into test (20%) and train (80%) subsets. To evaluate the effectiveness of machine learning classifiers, six different algorithms were used to predict the probability that a fatal cerebral haemorrhage is present on the given PMCT dataset. The types of binary classifiers were *k*-nearest neighbours, Gaussian naive Bayes, logistic regression, linear discriminant analysis, decision tree, and support vector machine. Each algorithm was implemented in Python (version 3.10) [18] with PyTorch (version 1.12.1) [20] and Python’s scikit-learn library (version 1.0.2) [21]. Parameter optimization was performed for each algorithm to identify the settings that would yield the best performance for the algorithms under consideration. These optimizations were based on the results from the testing subsets and the respective outcomes are summarized in Table 1.

**Table 1:** ML classifier parameter optimization

Classification algorithm	Parameters
*K*-nearest neighbour classifier	{'algorithm': 'ball_tree', 'leaf_size': 10, 'weights': 'uniform'}
Gaussian naive Bayes	{'var_smoothing': 1e-08}
Logistic regression	{'max_iter': 300, 'penalty': 'l1', 'solver': 'saga'}
Linear discriminant analysis	{'solver': 'svd'}
Decision tree	{'criterion': 'gini', 'splitter': 'best'}
Support vector machine	{'kernel': 'linear'}

The binary classification task was then executed utilizing the parameter configurations derived from the parameter optimization process. The classification algorithms were trained and tested 5 times, with each fold serving as the testing set once, whilst the remaining folds were used for training. This process was repeated to cover all possible combinations of training and testing sets. By using a stratified *k*-fold cross-validator, it was made sure that the percentage of samples for each class could be preserved in all folds (44–47% for the pathological cases and 53–56% for the healthy control cases, depending on the number of datasets within each fold). The pathological and healthy data classes were therefore always highly balanced in all folds. To derive a comprehensive and representative evaluation result, the average performance across the five-fold cross-validation (CV) iterations was computed. This approach provided a more reliable estimate of the algorithm’s generalization capability, considering the performance on multiple subsets of the data.

**Fig. 2:**
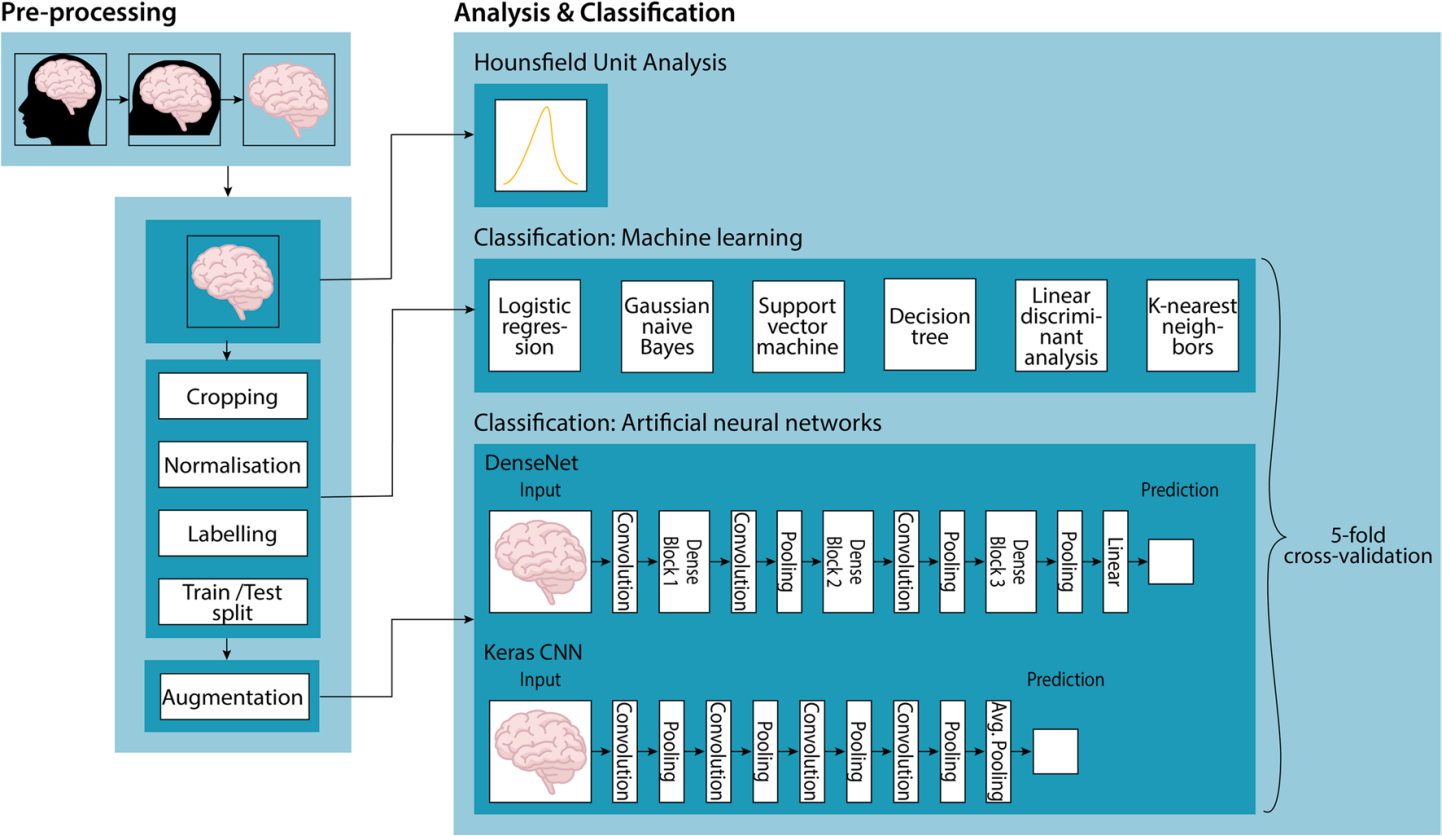
Graphical representation of the complete classification pipeline

Furthermore, two different 3D artificial networks were trained and evaluated. They differed in various aspects concerning the used framework or library, the architecture of the network, and the use of augmentations. The 3D convolutional neural network (CNN) model architecture was based on the publication from Zunair et al. [22]. It consists of four 3D convolution, maxpool, and normalization layers, followed by a binary classification. It was implemented with Tensorflow (version 2.0.0) [23] and the Keras library (version 2.3.1) [24] based on [25]. This model was trained with 200 epochs. The best results were achieved with a batch size of 4, a learning rate of 0.0001, and a dropout layer of 20%. The second algorithm was a 3D densely connected convolutional network (DenseNet), which is a network architecture with dense connections, whereas the connectivity pattern ensures maximum information flow between layers in the network [26]. The DenseNet model was implemented using PyTorch (version 1.12.1) [20] and the open-source framework Monai [27]. Thereby, the best results could be obtained with a batch size of 6, a learning rate of 0.0001, and number of epochs of 150, using the test subsets. The used activation function was the sigmoid function, which takes any value as input and output values in the range from 0 to 1. Both model architectures were trained with an image size of 128 px for each dimension and included various augmentations (rotation, elastic transform, random brightness, and contrast). The applied loss function was binary cross-entropy, and the optimization algorithm was Adam [28]. The whole dataset was split into a training (80%) and a test (20%) set. Both deep learning networks were trained on a Linux system (Cuda version 11.4 server) with a GeForce RTX 3090 graphics card. A five-fold cross-validation was applied with highly balanced samples in the training and testing sets of each fold, mirroring the methodology used for the machine learning algorithms. The obtained results of all machine learning and deep learning classifiers were compared regarding their sensitivity, specificity, accuracy, and precision. Additionally, the area under the receiver operating characteristic (ROC) curve was created for each algorithm. The complete pipeline is illustrated in Fig. 2.

## Results

**Fig. 3:**
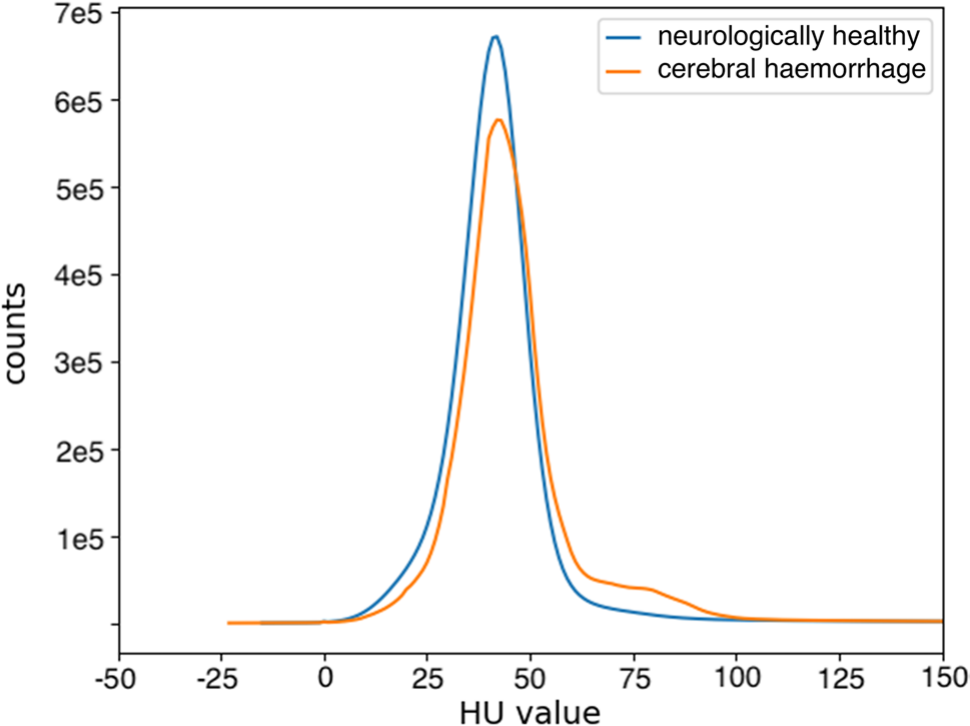
Histogram of HU value counts in pathological datasets with cerebral haemorrhage (in orange) compared to neurologically healthy cases (in blue)

The histogram illustrated in Fig. 3 is a representation of the distribution of the occurrence of HU values in the cases with fatal cerebral haemorrhage compared to the neurologically healthy control cases. For this analysis, the HU values were obtained from all pixels within the segmented 3D data for all cases. Thereby, only values are illustrated, which have a higher count than 100 and are in the range of − 50 to 150 HU. The overall distribution of the pathological datasets is slightly shifted to the right compared to the healthy data. Additionally, there is a distinct difference between the two graphs within the ranges of 40–50 HU and 60–90 HU.

**Fig. 4:**
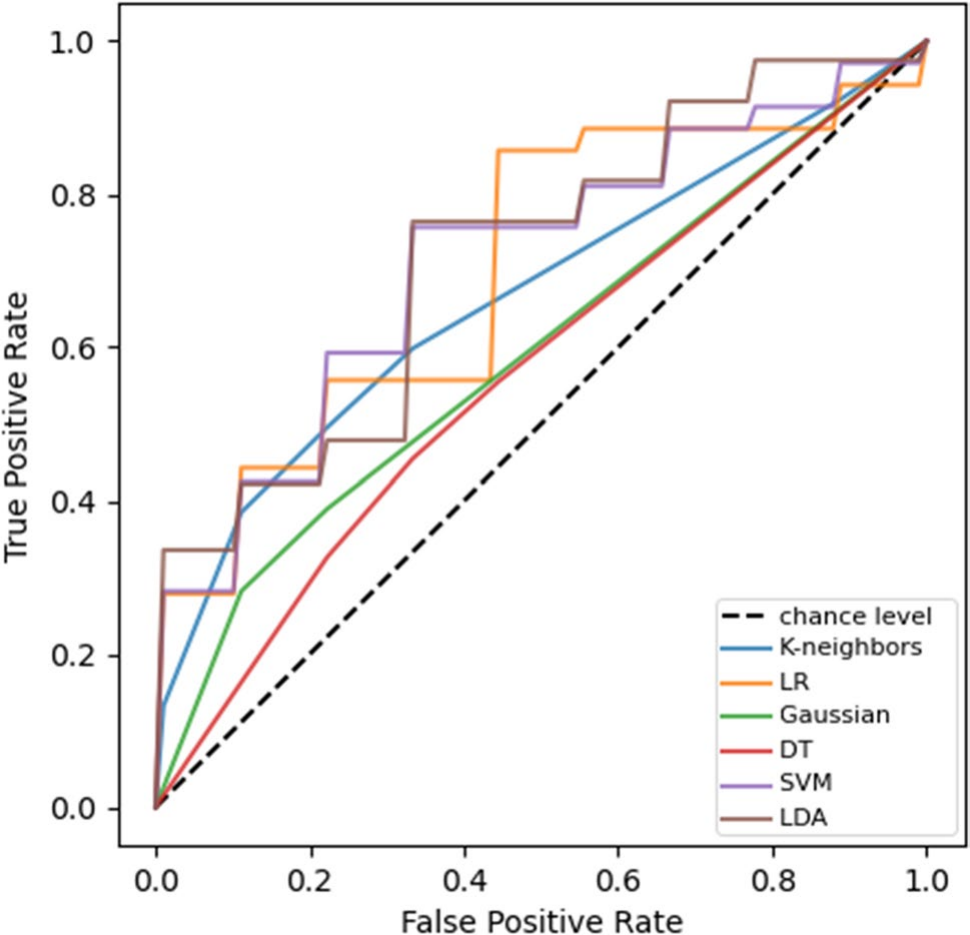
Average ROC curves of ML classification algorithms, where the black dashed line indicates an AUC of 0.5. *K*-neighbours = *k*-nearest neighbour classifier, LR = logistic regression classifier, Gaussian = Gaussian naive Bayes classifier, DT = decision tree classifier, SVM = support vector machine classifier, and LDA = linear discriminant analysis classifier. The curves appear either stepped or smooth depending on the nature of the classifier. The purple and brown lines have overlapping areas

A graphical overview of the results of the six different machine learning algorithms is illustrated in Fig. 4. The detailed mean classification results are further displayed in Table 2. The linear discriminant analysis, the support vector machine, and logistic regression algorithm achieved the highest AUC with 0.72, 0.71, and 0.70 accordingly. The poorest results were obtained by the Gaussian naive Bayes and decision tree algorithm with an AUC of 0.59 and 0.57, respectively. The best performing ML algorithm was linear discriminant analysis with a mean accuracy of 0.66. There were no significant differences in the classification results regarding the type, the location, or the size of the haemorrhage. The classification algorithms had more difficulties to categorize the pathological cases into the correct class, whereas only two healthy cases (in total) were classified wrongly by all classification algorithms.

**Table 2:** Detailed classification results of the six different ML algorithms (mean values)

Classification algorithm	Precision	Accuracy	AUC	Sensitivity	Specificity
*K*-nearest neighbours	0.98	0.61	0.67	0.59	0.86
Gaussian naive Bayes	0.82	0.63	0.59	0.62	0.64
Logistic regression	0.71	0.64	0.70	0.65	0.61
Linear discriminant analysis	0.76	0.66	0.72	0.67	0.65
Decision tree	0.69	0.59	0.57	0.61	0.55
Support vector machine	0.76	0.63	0.71	0.64	0.62

**Fig. 5:**
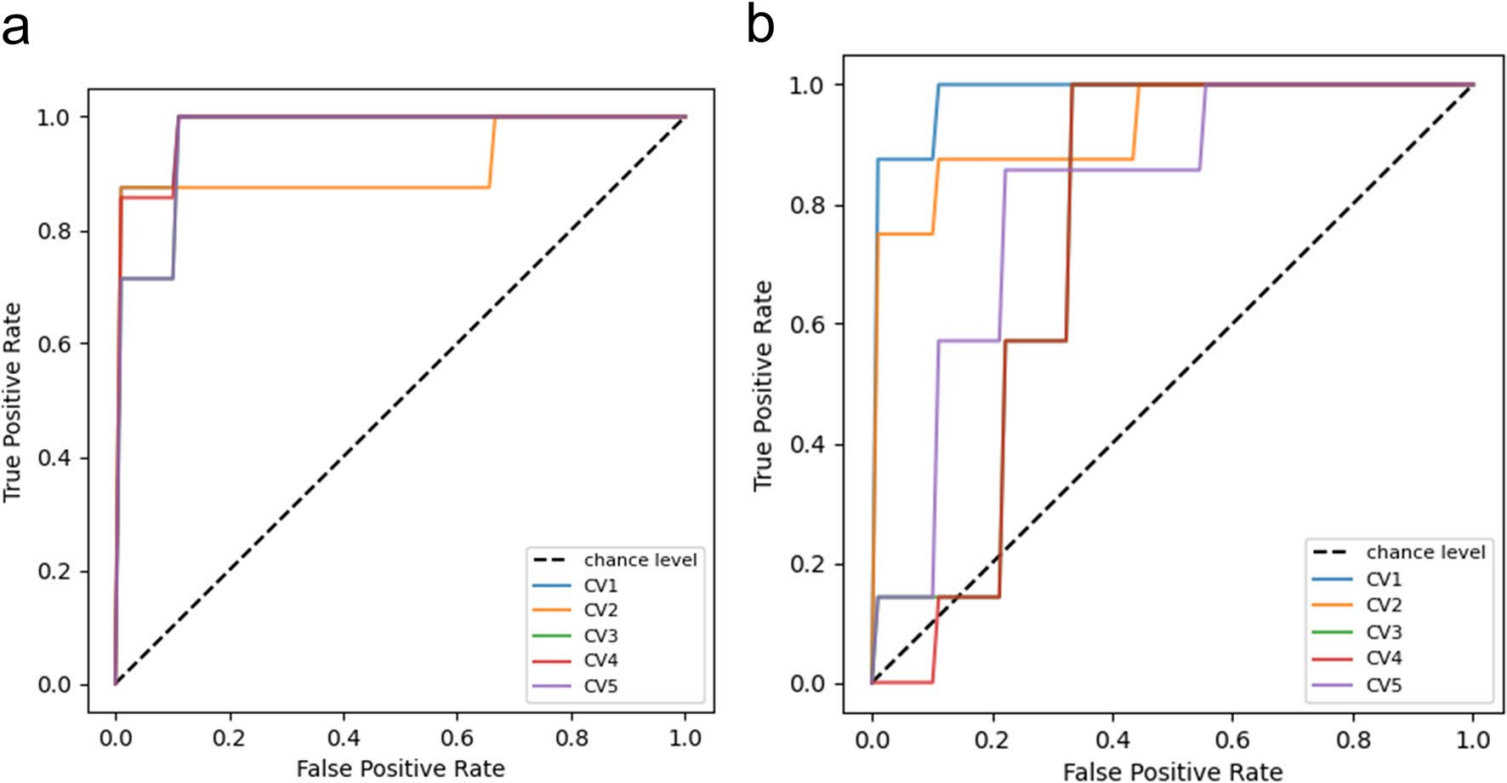
**a** ROC curves of the CNN classification network, where the black dashed line indicates an AUC of 0.5. The different coloured lines represent the AUCs of the five cross-validation classifications (CV1–CV5). The blue, green, red and purple lines are overlapping at most parts. **b** ROC curves of the DenseNet classification network, where the black dashed line indicates an AUC of 0.5. The different coloured lines represent the AUCs of the five cross-validation classifications (CV1–CV5). The green line overlaps with the red line and hence appears brown

A five-fold cross-validation was performed on both deep learning models (CNN and DenseNet). The results (mean values over all folds and corresponding standard deviations) for both networks are listed in Table 3. The CNN network achieved a mean accuracy of 0.94 and a mean AUC of 0.96 and demonstrated a higher mean specificity compared to its mean sensitivity. Furthermore, all folds yielded comparable AUCs, as illustrated in the ROC curves in Fig. 5a. The classification results for the DenseNet network were inferior to those of the CNN network. Overall, a mean accuracy of 0.84 and a mean AUC of 0.85 were achieved for DenseNet. The AUCs of the different folds of DenseNet showed more variation compared to those of CNN (Fig. 5b). As observed for the CNN network, the DenseNet model also exhibited a lower mean sensitivity compared to its mean specificity.

**Table 3:** Cross validation results for the CNN and DenseNet classifications (mean values and corresponding standard deviations)

Classification model	Precision	Accuracy	AUC	Sensitivity	Specificity
CNN	0.98 ± 0.05	0.94 ± 0.002	0.96 ± 0.03	0.91 ± 0.05	0.98 ± 0.05
DenseNet	0.93 ± 0.06	0.84 ± 0.07	0.85 ± 0.10	0.78 ± 0.17	0.92 ± 0.07

**Fig. 6:**
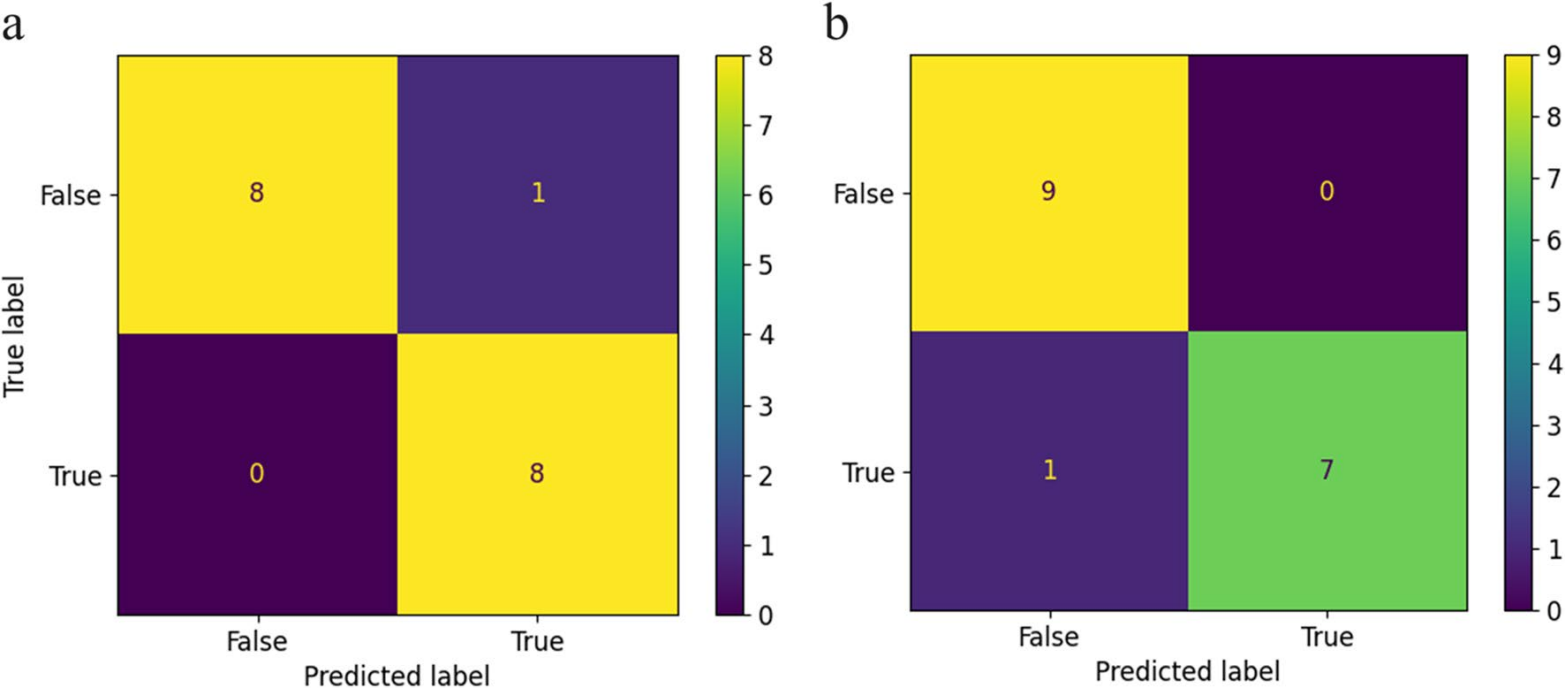
**a** Exemplary confusion matrix of one CNN classification cross-validation with one false negative (CV1). **b** Exemplary confusion matrix of the top-performing DenseNet classification with one false positive (CV1)

In Fig. 6, exemplary confusion matrices for both artificial neural networks are illustrated to provide an overview of their classification performance.

## Discussion

In this study, we developed and investigated a pipeline capable of automatically classifying cranial PMCT datasets as either pathological with a fatal haemorrhage or as neurologically healthy using different machine and deep learning models. In recent years, artificial intelligence has not only found its way into medical imaging, but has also revolutionized acceleration of imaging acquisition, image reconstruction, and image analysis amongst other aspects [29]. In general, machine and deep learning requires a large number of well pre-processed data and labelling to achieve accurate classification; however, collecting a sufficient amount of data still is a very difficult task in the medical fields [30]. The aim of our work was to use PMCT data from the database of our institute and to strongly reduce the workload for pre-processing by using the automated cranial segmentation algorithm by FSL, which showed very reliable results. The results are consistent with the publication of W. Breakey et al. [16] who also achieved robust segmentations of CT data with this algorithm.

Our exploratory data analysis did show a higher occurrence in the range of 60–90 HU for the pathological group with cerebral haemorrhage, which may be an indication of more coagulated blood (50–75 HU [31, 32]) and/or a subdural hematoma (65–100 HU until 3 days after death [31, 32]). On the other hand, the peak at around 45 HU observed in the healthy cases is higher compared to the pathological data, which may refer to the healthy brain tissue. This could suggest a difference between the two investigated groups and might show a useful starting point for machine learning investigations. However, these results may only give a hint on the potential presence of a cerebral haemorrhage.

For the classification of the datasets, six common machine learning algorithms (*k*-nearest neighbour classifier, Gaussian naive Bayes, logistic regression, linear discriminant analysis, decision tree, and support vector machine) were considered in the present work. Although nowadays mainly deep learning models are applied for such purposes, machine learning may still be worth pursuing to investigate simple classification possibilities first, especially when dealing with small datasets. The utilization of machine learning methods provided a first indication of the classification performance. However, none of the ML algorithms could provide a reliable classification result. The ML investigations also showed that the result was depending on the training and testing subsets of the different cross-validation folds. This points at the importance of applying additional data augmentation and having a large dataset at hand. In summary, the complex classification of PMCT images by simple ML algorithms was not suitable for obtaining robust results.

However, a different picture emerged for the deep learning models, where highly promising results were obtained with a mean accuracy of 0.94 for the CNN network. The CNN network, validated through a five-fold cross-validation process, exhibited consistent and reliable performance metrics, affirming its efficacy in handling complex patterns and feature extraction within the data. Contrastingly, the application of cross-validation revealed that DenseNet did not exhibit optimal performance for all subsets. Nevertheless, the results of the two artificial neural networks are comparable to previous analyses of head injuries based on antemortem CT data [9–11]. The study of Chilamkurthy et al. [10] achieved an AUC of between 0.86 and 0.97 for detecting five types of cerebral haemorrhages, whereas Jnawali et al. [9] achieved an AUC of 0.87 with their 3D CNN architecture.

So far, deep learning techniques have been extensively researched for different tasks based on 2D images; however, the number of publications including deep learning and postmortem data is still very limited [10, 33, 34]. One exception, worth mentioning, is the study of Garland et al. [35] that investigated fatal head injuries on postmortem CT and obtained an accuracy of 0.70 based on a small dataset with 25 cases of fatal head injury and 25 controls. In this study, cerebral haemorrhages were classified using 3D PMCT and two types of artificial neural networks. The superior CNN model was able to classify all testing datasets correctly, except for one false positive in four out of five folds and one false negative in one of five folds. Neither the PMCT images nor the data analysis gave any indication as to why these cases were misclassified.

To obtain robust and reliable results for the future, more data need to be collected and trained to strengthen the results of these networks. Based on the small amount of cranial data investigated in this work (36 pathological cases, 45 healthy cases), it was not possible to discriminate between different types of haemorrhages. However, as Baumeister et al. [1] stated, the discrimination of intraparenchymal and extraaxial haemorrhages might be relevant in regard to the manner of death. Another limitation of training a complex classifier on a small dataset is the potential risk of over-fitting, which could in principle lead to low performance and generalization power of the models [34].

## Conclusion

In this work, we propose a pipeline that enables the binary classification of fatal cerebral haemorrhages using 3D cranial PMCT scans. Especially the results of the artificial neural network CNN demonstrate that the automated detection of cerebral haemorrhages is possible with a high mean accuracy of 0.94, thereby highlighting the importance of selecting a neural network architecture tailored to the intricacies of the dataset. In the future, deep learning algorithms may be applied by forensic pathologists in forensic routine and might provide a helpful computer-assisted diagnostics tool supporting PMCT-based evaluation of cause of death.

## Data Availability

The datasets generated during and/or analysed during the current study are available from the corresponding author on reasonable request.
